# m-Health: Lessons Learned by m-Experiences

**DOI:** 10.3390/s18051569

**Published:** 2018-05-15

**Authors:** José Bravo, Ramón Hervás, Jesús Fontecha, Iván González

**Affiliations:** MAmI Research Lab, University of Castilla-La Mancha, Ciudad Real 13071, Spain; ramon.hlucas@uclm.es (R.H.); Jesus.fontecha@uclm.es (J.F.); ivan-gdiaz@uclm.es (I.G.)

**Keywords:** m-Health, human-computer interaction, frameworks, big data analytics

## Abstract

m-Health is an emerging area that is transforming how people take part in the control of their wellness condition. This vision is changing traditional health processes by discharging hospitals from the care of people. Important advantages of continuous monitoring can be reached but, in order to transform this vision into a reality, some factors need to be addressed. m-Health applications should be shared by patients and hospital staff to perform proper supervised health monitoring. Furthermore, the uses of smartphones for health purposes should be transformed to achieve the objectives of this vision. In this work, we analyze the m-Health features and lessons learned by the experiences of systems developed by MAmI Research Lab. We have focused on three main aspects: m-interaction, use of frameworks, and physical activity recognition. For the analysis of the previous aspects, we have developed some approaches to: (1) efficiently manage patient medical records for nursing and healthcare environments by introducing the NFC technology; (2) a framework to monitor vital signs, obesity and overweight levels, rehabilitation and frailty aspects by means of accelerometer-enabled smartphones and, finally; (3) a solution to analyze daily gait activity in the elderly, carrying a single inertial wearable close to the first thoracic vertebra.

## 1. Introduction

There is no doubt that the increasing use of smartphones makes them the most widespread electronic device around the world. In fact, current growth rates show us a greater number of mobile devices than world inhabitants. Their miniaturization, process, storage, and communication capabilities are accelerating the deployment of systems and applications, especially in the area of m-Health. Furthermore, embedding sensors into mobile devices enables new generations of health applications. Accelerometer, gyroscope, GPS, camera (back and front), microphone, NFC, touch screen, Wi-Fi, Bluetooth, and other embedded features suggest that smartphones are excellent devices for supporting applications in many domains like healthcare [1], environmental monitoring [2], etc. Thus, this represents an evolution from traditional health (and later telemedicine) to wireless and mobile systems, which are cost-effective, flexible, and efficient [3].

Before the emergence of m-Health term, the concept “unwired e-med” was introduced as a movement from telemedicine to wireless and mobile Internet applications [4]. Subsequently, wireless communications, networks, and wearable systems have advanced significantly [5,6]. These improvements have had a substantial impact on e-Health Health (i.e., healthcare practice supported by information and communication technologies [7]). The benefits of wireless technology have been illustrated in the literature [8]. Using this technology, information about patients is now easily accessible to healthcare staff, independently of their location. This information includes patient records, lab analysis, pharmacy information, and more. Traditionally, wireless is associated with “biomonitoring” by including parameters such as heart rate, blood pressure, blood oximetry, and other vital signs. Other areas include motion monitoring to detect and analyze falls, activity recognition, and location tracking. In this sense, instrumental gait analysis also called quantitative gait analysis has become an important research line in mobility domain over the last few years. Gait analysis performed in an objective manner is a reliable screening tool to quantify physical performance and it is being used as a functional indicator in frailty assessment [9], as a tool for enabling physical recovery monitoring in rehabilitation therapies [10] or as a predictive indicator to detect cognitive disorders [11], among others.

m-Health has been defined as “medical and public health practice supported by mobile devices, such as mobile phones, patient monitoring devices, personal digital assistants (PDAs), and other wireless devices” [12]. Another definition of m-Health is “mobile computing, medical sensor and communication technologies for health-care [13]”. As an evolution of the concept, this author defines m-Health as “emerging mobile communications and network technologies for healthcare systems” [14]. Norris et al. [15], in a study about m-Health sustainability strategies, concludes that m-Health plays a crucial role in healthcare. The authors define a strategy for selecting useful applications and the development of a set of principles and rules to support innovation and sustainability.

An important area of m-Health is aging. People are living longer, with the estimation that there will be about 1.5 billion people aged 65 or older by 2050 [16]. The impact of a demographic change of this magnitude represents an urgent and immediate health challenge for governments worldwide. To deal with this, strategies like the Ambient Assisted Living Joint Program [17] aims to create better living conditions for older adults and strengthen industrial opportunities in Europe through the use of information and communication technology (ICT). m-Health holds a promise for improving health with independence and cost-effectiveness, transforming the traditional healthcare system by means of education, motivation, and connectivity.

In this context, ensuring personal wellbeing through smartphone applications in an increasingly aging population requires monitoring multiple human behaviors in physical, mental, and social dimensions [18]. In fact, it is possible to infer a range of behaviors on smartphones in real time so that users can receive feedback of their daily activities while managing their health. This kind of applications can be found in many platforms and operating systems, as well as application stores like Apple App Store, Google Play and the Microsoft Store [18]. However, the majority of current m-Health applications are focused on specific health dimensions like monitoring of physical activities, stress, and diet [19,20,21,22] and they require a high degree of explicit interaction and cognitive effort to use them and provide information useful to infer in user behavior. It is crucial to overcome barriers for monitoring different daily behaviors by minimizing user effort and providing universal access. In this sense, human-mobile interaction has to be addressed easily and, if possible, in a “disappeared” way. In addition, inferring human behavior through embedded sensors may enable long-term monitoring for wellbeing applications.

In this work we present some experiences by using smartphones, wearables and wireless communications in health environments. We have classified them in three domains: clinical information management, monitoring of vital signs, and physical activity. Apart from smartphones, we made use of three technologies: Near Field Communication (NFC), inertial measurement units (IMUs) both integrated in phones or in dedicated devices and Wi-Fi or Bluetooth communication technologies.

## 2. Related Works

In this section, we divide related works into three groups. The first one covers managing patient care, nursing education and mental health regarding cognitive impairments and behavior changes. The second describes approaches for mobile monitoring of vital signs. Finally, the third presents several proposals in terms of physical activity recognition and gait analysis.

According to the first group, there are some approaches studied in the literature. Wyatt [23] showed a selection of several proposals in this field, which include drug database use, medical calculators, medical references, and personal records access. Regarding the latter, Choi et al. developed the MobileMed system [24] with the purpose of integrating the distributed and fragmented patient data across heterogeneous sources to make them accessible through mobile devices. In this sense, Burdette, Herchline, and Oehler [25] studied the advantages and disadvantages of using devices such as smartphones in clinical practice.

Concerning nursing education, Phillippi [26] proposes that the use of smartphones by students can reinforce learning at any time or location. Thus, they can access educational materials and guidelines during clinics, classes, or medical conferences. Also, Garret [27] proposes the e-portfolio tool. With this, the use of mobile technologies improves clinical learning, practice and, clinical and translation knowledge.

In terms of mental health in cognitive impairment environments, Luxton [28] provides an overview of the use of smartphones in behavioral healthcare. In this work, the limitations, practical issues, and recommendations are studied. Helal [29] presents a research project composed of three assistive applications to reduce the demand of elderly care and effort while performing daily tasks. In other particular contexts, like caring for those with Alzheimer’s disease, Zmily et al. [30] presented an application called ADcope that uses smartphones during the rehabilitation process. It includes modules such as memory wallet, calendaring, and dementia exercises. There is also another module supported by NFC technology by means of tags placed in drawers and doors. There are also proposals that aim to assist people with cognitive impairments combining mobile technology with augmented reality [31] with videogames [32], and with affective Avatars [33].

With respect to the second group of related works, focused on monitoring of vital signs, some approaches have been collected from the literature. In Rodriguez et al. [34] an m-Health application for heart rate and electrocardiography (ECG) monitoring is studied. Authors discuss whether this kind of solutions should be divided into three categories according to how vital data are acquired and processed. The first category involves recording signals and taking actions off-line. The second performs quasi real-time remote processing, and the last aims to provide on-line real-time processing from the mobile device itself. For example, Holter systems belong to the first category analyzing ECG for 24–48 h and afterwards by doctors. Instead of offering real-time monitoring, there is no immediate action when alarming situations occur. Other approaches in the literature promote the use of the mobile phone as a transceiver node gathering data from heterogeneous sensors and transmitting them to remote dedicated servers and cloud services. Some examples of this category are Cardio Control [35] (ECG monitoring), MediSense [36] (glucose monitoring), and MORF [37] (configurable array of heterogeneous sensors to monitor various vital signs). Particularly, the MORF approach was tested by receiving data from pulse, oxygen saturation (SpO_2_) and blood pressure sensors on the mobile phone which acts as an intermediary node between the sensors and the remote servers. All of the above-mentioned applications, corresponding to the second category defined by Rodriguez et al. [34], have several drawbacks because of the limitation of not reacting immediately in the place where the signals are received.

Finally, the third category includes vital sign monitoring applications that get data, store and process them locally. Several of these applications provide in-situ real-time alarms and/or send notifications to remote control centers. There are some examples, such as WAITER [38] that proposes wearable sensors for collecting vital signs and transmitting them by Bluetooth to the mobile phone. Also, the monitoring of heart rate to avoid heart attacks using wireless sensors and smartphones is addressed by Gay et al. [39].

Other relevant examples of systems to monitor vital signs in the literature are the following: the ICare [40] system proposes mobile health monitoring for elderly people by using wireless sensors and smartphones. This monitors remotely elders anytime, anywhere, and has a tailored service for each personal health condition. Petersen et al. [41] study a modular framework with m-Health applications. Among the different use cases evaluated, they find a simple and lightweight finger oximeter connected to the smartphone through its audio minijack provides an easy and non-invasive solution for measuring the blood oxygen concentration regularly. This is an interesting approach which opens up the possibility of daily monitoring of oxygen saturation enabling long-term assessments for prediction and treatment in a wide range of diseases. Other framework approaches can be seen in research conducted by Poon and Zhang [42]. This work defines four layers: personal, home, community, and hospital. Other aforementioned approaches also present their corresponding frameworks. Finally, in order to detect pulmonary anomalies, home spirometry is an interesting approach for the medical community. The SpiroSmart [43] is a low-cost solution for performing home spirometries through the smartphone’s microphone. After the revised proposals, it is important to mention the idea of generalizing by using vital sign frameworks.

The last important group of works according to our initial classification corresponds to monitoring of physical activity and gait analysis. First, we have to draw attention to the literature concerning accelerometers and gyroscopes as the main body sensor for receiving human movement. Kern et al. [44] presented a set of two or three single-axial accelerometers placed on several body segments for detecting static and dynamic activities. Similarly, using the same kind of accelerometers connected to a system called Vitaport [45], the recognition of 13 physical activities was achieved. This work focuses on rehabilitation treatments. Other authors have also investigated the discrimination of postures and movements using a minimal set of single-axial accelerometers [46].

The emergence of triaxial accelerometers increases accuracy, making them ideal components to recognize any physical activity. Maki et al. [47] present a proposal for activity recognition in elderly people at home, by means of a triaxial accelerometer placed on the chest, six passive filters, a microcontroller, and a mobile phone. Other research [48] recognizes physical activities using a triaxial accelerometer placed near the pelvic region. Trunks accelerations from a triaxial accelerometer located at the L3 vertebra (lower trunk) are used by González et al. [49] for heel-strike and toe-off event demarcation in quantitative gait analysis sessions. Same gait events are segmented by López-Nava et al. [50], although two body-worn triaxial accelerometers are attached to the ankles in this case. Finally, a mobile sensing platform is presented in work by Choudhury [51]. The device developed includes a triaxial accelerometer, among other embedded sensors such as triaxial gyroscope, microphone, light phototransistor, etc.

There are several approaches regarding the use of an accelerometer embedded into mobile phones or smartphones. Anguita et al. [52] present a proposal for activity recognition through smartphones with built-in accelerometers. A Modified clustering algorithm based on Super Vector Machine (SVM) is used. Specifically, the SVM clustering is adapted to exploit fixed-point arithmetic for computational cost reduction cost. In Dernbach et al. [53], a solution for simple and complex activity recognition is presented. Locomotion activity and other, more complex actions, like cooking and cleaning, are formulated by using a combination of the embedded accelerometers and gyroscopes from a single smartphone.

Another important area is fall detection. An online location support system by using mobile phones is studied by Yavuz et al. [54]. Also, the PerFallD [55] develops a platform for pervasive fall detection by combining detection and communication components, including mobile phones. Finally, Abbate et al. [56] system recognizes falls and sends notifications to caregivers for help.

The solutions presented in the last two paragraphs have to use the embedded accelerometers and gyroscopes in the mobile phone or smartphone but not distract the users’ attention. Thus, it is convenient to run these applications in the background, allowing users to manage other smartphone functionalities. At this point, it is important to highlight that all of the above-mentioned approaches determine the use of mobile devices through managing information into the built-in displays. In addition, it is well known that these devices are restricted and resource-limited for addressing all the processing and required interactions involved in acquiring and managing clinical data. For this reason, it should be convenient to adopt and promote the use of additional embedded technologies, dedicated devices and standalone servers to complement the smartphone’s role. It is essential to provide new ways for clinical data acquisition and human-device interaction; and also, enabling powerful data reasoning services to extract relevant clinical information.

## 3. Technologies for Supporting MAmI m-Health Approaches

Taking into account all the experiments and works carried out in MAmI Research Lab and regarding the three groups previously mentioned in the related works section, in this section, we are showing the two main technologies used. Apart from the own mobile phone or smartphone, as well as their communication capabilities, we have to mention NFC and accelerometer sensors.

### 3.1. Near Field Communication (NFC)

NFC is a short-range, wireless connectivity technology developed by Philips and Sony in 2002. It is a combination of RFID and interconnection technologies. This technology consists of two elements: the initiator for beginning and controlling the information exchange, and the target for replying the initiator’s request.

**Figure 1:**
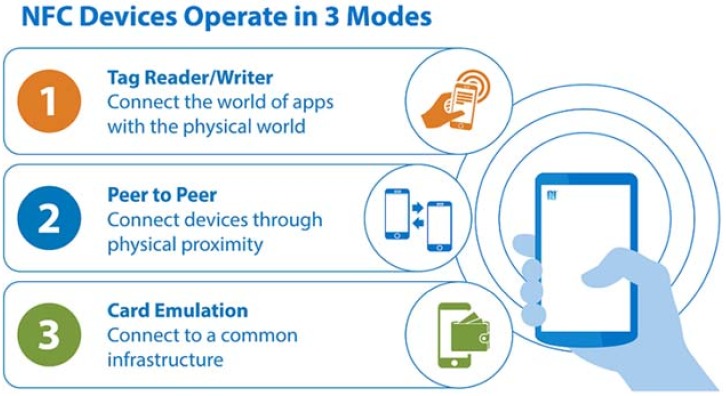
NFC operation modes.

In an NFC system there are three modes of operation based on different ISO standards as Figure 1 shows: (1) in tag reader/writer mode, NFC-enabled devices read and store information from NFC tags embedded in smart posters, displays, etc.; (2) in peer-to-peer mode, two NFC-enabled devices can communicate with each other to exchange information and deploy services; (3) and finally, in card emulation mode, NFC-enabled devices act like passive tags or smart cards for example to perform transactions such as purchases, ticketing or transit access control, with just a touch.

### 3.2. Accelerometers

An accelerometer is a mechanism used to measure accelerations and gravitational forces. The technological growth in mobile computing and the development of micro-electromechanical systems (MEMS) allow the integration of accelerometers in small devices like smartphones [57] and those from the Internet of Things (IoT) approach. The main aim of accelerometer sensors is to recognize movement patterns in object which have the sensor embedded. All accelerometers have the following features:

- Number of axes: The accelerometer can gather movement data from one, two, or three coordinate axes. Thus, there are single-axial, bi-axial, and tri-axial accelerometers.
- Amplitude range: Defines the minimum and maximum values to be reached by the sensor output.
- Sensitivity: Represents the accuracy of the accelerometer sensor.
- Frequency response: The frequency interval in which the accelerometer detects movement.
- Operating temperature: Ambient temperature range of the sensor to ensure a correct operation. Reference values are usually between −40 °C and 85 °C.
- Devices with accelerometers can be focused on gathering of movement data (specific purpose) or they can be general-purpose devices. The first ones consist of small devices, which include an accelerometer sensor and the necessary logic to collect data to establish communication with external sources. Meanwhile, the general-purpose devices usually have several sensors and functionalities because these devices are not only focused on gathering movement data. Currently, the best-known general-purpose device is the smartphone.

An appropriate integration of these technologies will lead to systems and services to acquire data in a natural and non-intrusive way to the end user.

### 3.3. Gyroscopes

Gyroscopes are another kind of MEMS sensors that provide the rate of change of angular position over time measured in degrees per second. This measurement is known as angular velocity and it is used to estimate the gyroscope’s relative orientation (tilt) with regard to fixed rotation axes. Orientations angles are estimated by integrating angular velocities. One of the problems of this integration process is that signal noise will also be integrated together with the real angular velocity magnitudes. In this sense, gyroscopes have their own limitations (in the form of a shifting bias/offset) when the device is not in perfect static conditions. Gyroscope bias is specially affected by temperature changes. These conditions lead to what is known as drift.

Although the drifting is very slight, when dealing with integration, even smallest bias will cause the integrated signal (tilt/orientation angle) to grow substantially. In this scenario, the combination of accelerometers and gyroscopes through Sensor Data Fusion algorithms helps to minimize the gyroscope propagating error getting better orientations angles. For this reason, accelerometers and gyroscopes are commonly packaged together into an Inertial Measurement Unit (IMU), which makes up a standardized base-line configuration for an inertial device.

## 4. Case Studies

The experience of the MAmI Research Lab in m-Health monitoring has been varied. In this section, we detail experiments that use the NFC technology for promoting the management of health record information. In addition, nursing care and training, Alzheimer’s care support, and mobile prescriptions are shown in detail. Besides, a framework for multi-monitoring vital signs is presented. Some proposals for activity recognition by means of accelerometer-enabled smartphones focused on childhood obesity, rehabilitation, and frailty, are also presented. Finally, we describe an infrastructure for quantitative gait analysis that uses a set of wearables intended to acquire inertial raw data from the trunk of elderly people in an assisted living environment. Specifically, linear accelerations and rotation angles from the trunk of each elder are processed to demarcate gait events and to compute derived gait parameters such as gait variability.

Table 1 shows a summary of these works with some information about pros and cons of each, before the detailed explanation.

### 4.1. Nursing Care

Nursing care in hospital wards is an excellent scenario to include NFC capabilities. NFC tags are placed in/on rooms, beds, patients, treatments, and so on. Thus, it is possible to manage patients’ nursing information by only using the smartphones with simple interactions. For example, nurses only need to touch the patient’s wrist in order to identify prescribed drugs and doses, as well as pending analysis, clinical tests, etc. All of the above mentioned uses make it possible to reduce the time taken to manage the patient’s information in terms of nursing care.

**Figure 2:**
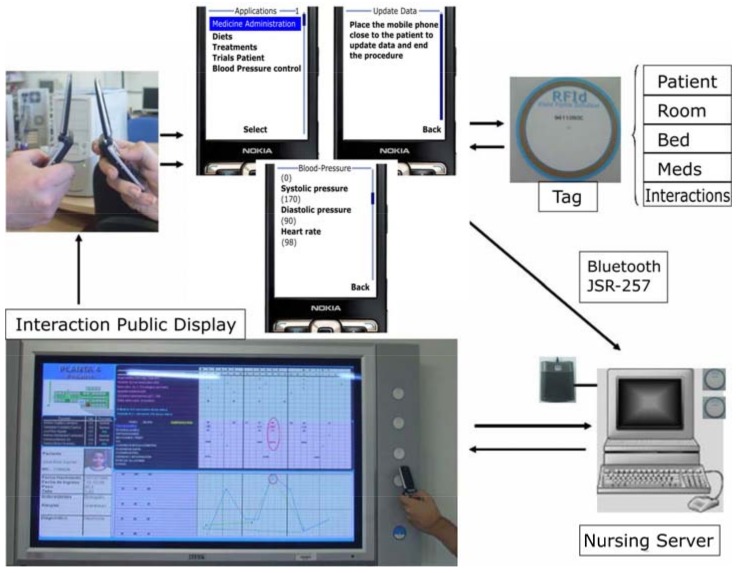
The nursing care cycle: A system prototype.

We have identified the “Nursing Cycle”, which can be seen graphically in Figure 2, where we have structured the different tasks that nurses have to manage in order to store patient data by only touching tags [58].

In the top left of Figure 2, nurses exchange information about patients with NFC-enabled mobile phones or smartphones. The schedule is transmitted from the nurse on duty to the incoming nurse, along with all needed information, such as the task classification by the time of the next shift. In addition, an alarm sounds when patients need particular care tasks. At present, nurses manage this information through forms that have to be prepared for each patient and shift. Following the cycle from left to right, in the next step, represented with some screens, nurse can control and visualize diet aspects, blood and urine tests among others. The third step, on the top right of the figure, represents different data that can be stored in NFC tags of patients’ wrists, rooms, beds or medicines. Also, smartphones of nurses can interact with a larger display by means of NFC tags to show detailed information of the nursing schedule. A server machine is responsible for attending requests from smartphone—display interactions.

All the tasks to be performed by nurses with our NFC system are shown in part (a) of the Table 2. It includes the place to develop the task, NFC tags with which nurse interacts, and the provided awareness for each one. In part (b) of the Table 2, collaboration process between nurses and physicians is shown. Similarly, it describes the place, NFC tag and process to carry out. Finally, part (c) of the Table 2 shows the content for each kind of NFC tag with the possibility to deploy a specific process (including awareness aspects). Patients’ tags contain all the information needed for nursing care, which means drugs prescribed and their doses, blood/urine tests (control of requests for these), diets, wound dressings, data monitoring, and the contraindications of the medication. Bed tags store useful data of each patient, such as identification, pending tests, and diet. The room tag contains the patient’s identification and other useful information. The tags for medication store specific information for every patient, with support for the control of his/her administration and the corresponding preparation in the nurse’s cabinet. Finally, the interaction tags can offer a way for nurses to store/show information in a large display.

Apart of the NFC adaptability in hospital environments, one of the most relevant works associated with this proposal is the visualization service through “Visualization Mosaic” [59,60]. In this, nurses are available to manage patients’ information by only touching the interaction tags placed on the right of the large display. Different parts represent the data of each patient and their evolution. The mosaic consists of different pieces of a puzzle showing the hospital ward map, a list of patients each nurse is looking after, general data of each patient, his/her particular nursing information, and evolution charts. These are generated automatically when the server receives the information, once again, only by touching tags in order to store the patient record from the caring tasks carried out. Finally, this display facilitates the collaboration between nurses thanks to its interaction tags in the shift process.

With this experience we tried to reduce the burden of managing patient records and information in a hospital ward by nurses. We have transformed the traditional methods of filling out forms with simple touches. This allows an easier information management so nurses can concentrate on caring for patients rather than handling data.

Non-mentioned aspects regarding interoperability, security and privacy, have been contemplated by including a framework called MoMo (detailed in Section 4.5) in a general solution called the Talisman + project [61].

### 4.2. Nursing Training

Another nursing-related study carried out by MAmI Lab was dedicated to performing nursing practices by using NFC technology in Nursing Schools. Students used a set of NFC tag panels, NFC bracelets, and NFC mobile phones to simulate different actions during a nursing routine. An NFC reader is connected to a computer, and the desktop software updates and downloads the corresponding nursing actions associated with each nursing student into the mobile phone.

The interaction between the NFC mobile phone of the student and any NFC tag or bracelet makes possible the deployment of a mobile application that runs the corresponding service. 

The aim of this proposal is to simulate the main actions carried out during a nursing routine. Only the use of NFC technology is necessary to monitor every action performed on the patient. These actions are grouped in services. The following services were identified [62]:

- Patient and nurse identification. Based on the roles assumed by nursing students, each participant is identified with an NFC tag (nurses) or an NFC bracelet (patient).
- Shift process between nurses. Supported by the NFC reader and the desktop application, two nurses can exchange their schedules as a typical shift process.
- Management and visualization of patient tasks and records in the desktop application. When a nurse interacts with the NFC reader, the desktop application shows the actions needed for the patients in that nursing routine.
- Monitoring of medication administration. The nurse can monitor the administration of drugs to a specific patient, interacting with the patient bracelet and the appropriate tag through the NFC mobile phone.
- Vital sign monitoring. In the same way, a nurse can register a new vital sign measure (e.g., blood pressure or temperature) related to a patient, touching his bracelet and the corresponding tag of a NFC tag panel (with representative icons related to variables and tests to be controlled).
- Registration of clinical tests. Another registration task corresponds to the clinical test registration. It monitors the performance of clinical tests to a patient, also by using the NFC tag panel.

**Figure 3:**
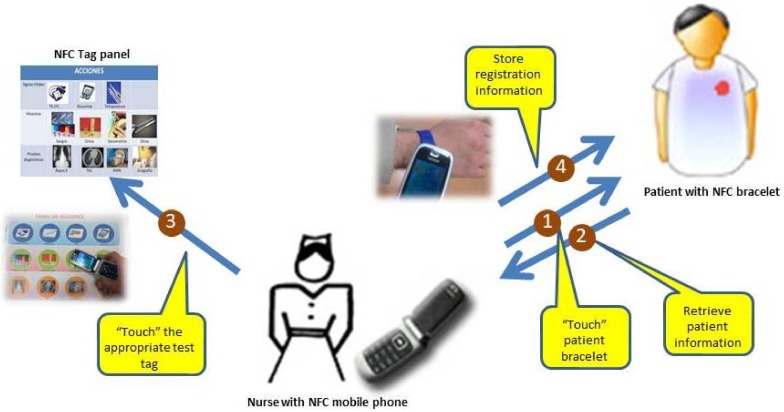
Example of using NFC nursing system: Steps for clinical test registration.

The NFC mobile phone interacts with the patient bracelet and the related NFC tag of the panel. Figure 3 shows an example of NFC interaction between the mobile phone and the tag panel for a clinical test registration. The stages to perform this task are presented in the figure (see steps 1 to 4).

This system was deployed in a nursing school in Ciudad Real and Albacete (Spain) to evaluate the integration of NFC technology in a nursing environment. Sixty-eight nursing students and eight teachers participated in this experiment. Some individuals assumed the role of nurses and others were patients. The nurses followed a simulated nursing routine of specific actions with several associated patients, using an NFC mobile phone and the reader connected to the desktop application to upload and confirm the task performed.

Before the experience, the participants did not know NFC technology; however, at the end of the experiment 71% of participants believed that the integration of these technologies would improve time, productivity, and their own nursing experience. Furthermore, the need to anticipate an improvement in nursing care poses a new challenge in the education of future nurses, and the use of non-intrusive systems, through simple interactions, encourages the technological adaptability among users. NFC and touch interaction is a good alternative as we have seen in this section. The integration of these kinds of systems improves student knowledge about mobile technology handling in a hospital environment.

### 4.3. Alzheimer Caregivers’ Support

Alzheimer patients need special attention by caregivers who cannot distract themselves from caring while managing records at the same time. Thus, we have adapted the NFC technology in order to support caregivers in an Alzheimer’s day center [63]. Patients go there every day from 9:00 a.m. to 4:00 p.m. During this time, relatives are relieved from the continuous daily care of Alzheimer’s patients. In this center, patients have to do a few tasks every day, such as:

- Rehabilitation/Physiotherapy.—This is to observe patient behavior and to promote activities by means of several physical exercises. In these activities, caregivers need to know about recent injuries caused by falls or domestic accidents. In addition, information about patient profiles and their physician’s recommendations are also relevant.
- Therapy.—This is the main activity that seeks to reinforce the patient’s memory by the recognition of relatives and objects. Patients are asked about details of their families, e.g., the names of children, wife or husband, parents, and so on. In object recognition exercises, patients are asked to recognize pictures with the aid of their therapists. There are also a few games to reinforce memory included in this activity.
- Handwork (Occupational Therapy).—This consists of cutting out drawings painted on cardboard, sticking different parts together, and finally creating figures.
- Visual.—Once a week, patients watch films and documentaries in the projection room. Meanwhile, the staff manages the weekly information in order to draw recommendations for the families in their weekend care.
- Lunch.—This is considered another important activity because it includes the patient’s behavior when eating. Patient attitudes and reactions at lunch-time, such as rejection of food, have to be monitored by care-assistants. Facts such as menu acceptance, refusal to eat, and affinities with other patients or fights are important aspects of daily life at the center.

**Figure 4:**
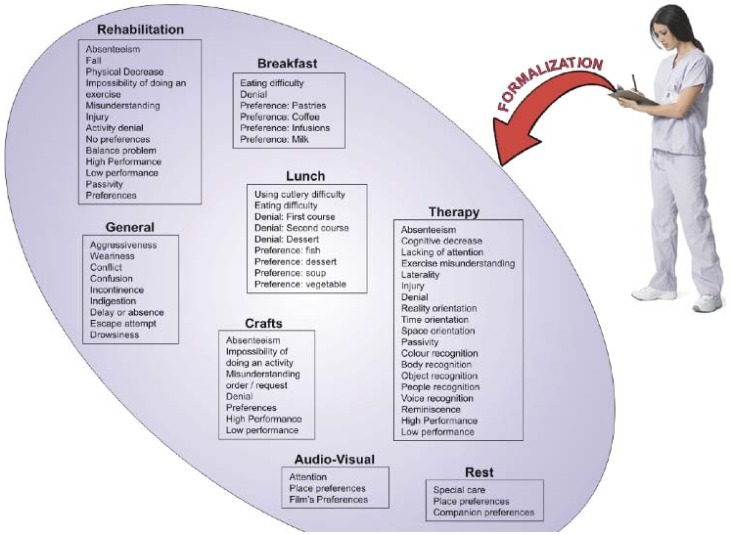
Classification of incidences at Alzheimer’s day center.

It is important to mention that during the previous activities, incidences that occurred have to be registered in a notebook. Noticeably, many incidences are also transmitted informally just by speaking. This means that some things are often forgotten since there is no corresponding annotation in the notebook. For the aforementioned mentioned, it is important to formalize the information regarding incidences. In Figure 4 the classification of incidences is shown.

**Figure 5:**
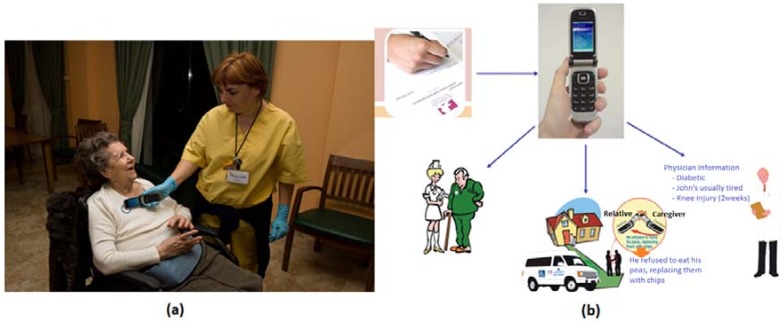
Tagging incidences (**a**) and automatic information management (**b**) with NFC.

From this classification, we have obtained the most relevant incidences applicable to the center of evaluation. In addition, each center could add its own incidences through a common server interface or even a website. This is a concept that we have called meta-incidents. Once the incidents have been formalized, a form is offered to the user to complete in a consistent way. With NFC technology it is possible to simplify this task. In combination with a mobile phone, NFC technology allows a user to “insert” the incidence in the patient NFC tag as is shown in Figure 5a. Other caregivers, therapists, or relatives can then access this easily.

At this way, users should receive the personalized data. Physicians are able to receive clinical information, and relatives and caregivers also receive it in an appropriate way (see Figure 5b).

In this kind of environment this approach to NFC adaptability significantly improves the caring services received by patients and the daily activities of caregivers at the day center. Only the adoption of natural interaction mechanisms can encourage the use of emergence technologies for caregivers.

### 4.4. Mobile Prescriptions

In this experience, we try to support daily activities of elderly people. With natural and closer interactions, patients can request medicines from their own homes. The current process is difficult, usually beginning with calling the medical center to arrange an appointment with the family doctor and subsequently going to the medical center to wait to obtain the prescriptions needed. Then, patient goes to the pharmacy to acquire the prescribed medicines [64].

In our case, simply interacting with NFC tags on medicine boxes changes this arduous process. When a patient interacts with these NFC tags, the family doctor receives the request in his system. Then, the doctor prescribes the requested medicines by sending a message to the nearest pharmacy in charge of delivering medicines to the homes of elderly patients.

**Figure 6:**
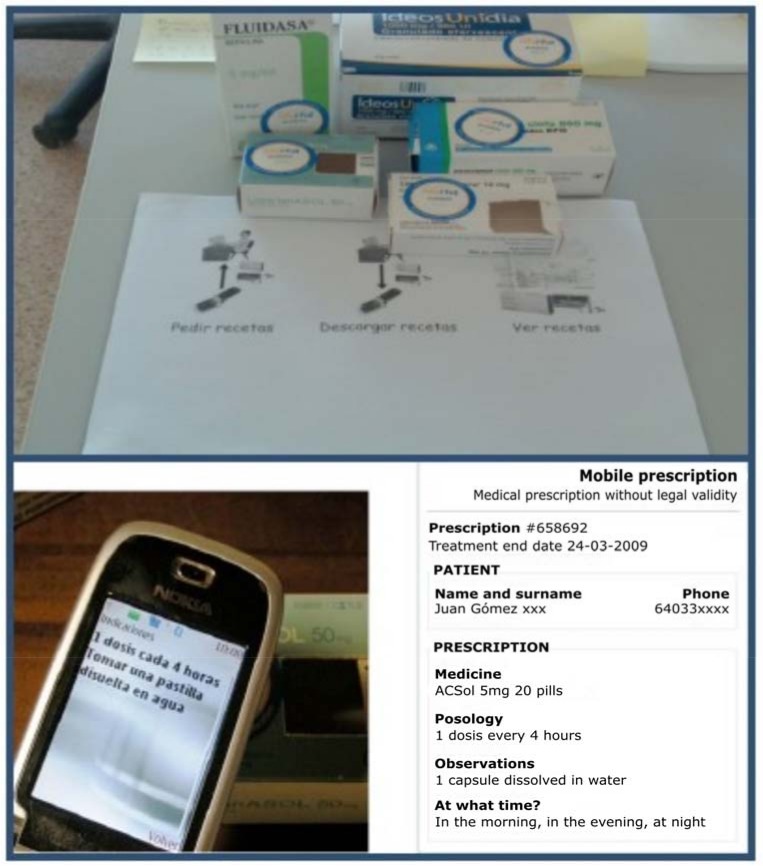
Panels and NFC-Enabled Medicine Boxes (**above**). NFC interaction (**below**).

Figure 6 shows the NFC-enabled process for prescriptions. By means of a panel, it is possible to begin by touching a control tag. Then, patients touch the needed medicine boxes and, finally, another control tag is in charge of sending the requested medicines to the doctor.

There are patients who need to take many medicines. In these cases, confusion and oversights can lead to a wrong medical treatment. For this reason, NFC tags attached to the medicine boxes are also used to support this issue with personalized information about doses and frequency of these. For example, this approach can offer alert messages in the case of a past or upcoming expiration date of the medicine, or if the end of the treatment has been reached. There can also be an alert sent when it is time to take medicines following the family doctor’s prescription.

In this approach, we have adapted the NFC technology in order to transform the usual method of obtaining prescriptions of care-dependent people to help them to achieve wellness and improve quality of life.

### 4.5. Vital Signs Multi-Monitoring

One of the main challenges of m-Health is to promote the easy day-by-day life of people with chronic diseases. Our proposal in these terms is a software architecture called MoMo which allows the semi-automatic generation of personalized applications to monitor diseases using mobiles devices (health monitoring) [65,66]. This architecture provides continuous patient monitoring to improve the communication between patients and doctors.

**Figure 7:**
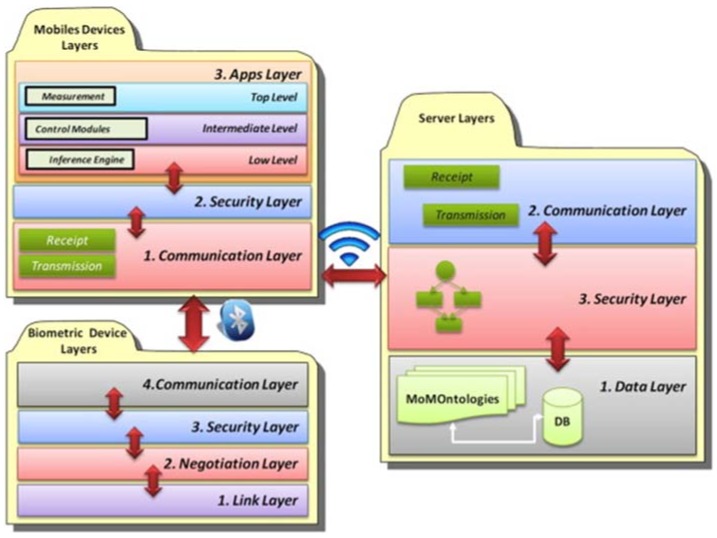
Layers overview of MoMo framework.

In order to facilitate the application generation, the data model has been formalized using ontologies. The ontological model includes the patient’s profiles, where the personal details of the patient are specified, and the definition of the modules for the mobile phone. Using the ontological definition, the infrastructure to generate is distributed into layers to organize the functionalities. These layers are shown in Figure 7 and include details about the layers for the mobile applications, the layers for the server support, and the layers to enable communications between the mobile device and the biometrics devices. Furthermore, to improve the communication between patients and doctors, this framework also provides features for continuous patient monitoring and support of an automatic architecture for the individual profiles of each patient, and self-control and education modules for their condition. The three important elements in this framework are the patient profile, module definition, and the communication architecture. The first is responsible for storing patients’ characteristics. The second defines all modules that will be deployed by means of patterns and relations between them. Also, this element takes part in data collecting for the individual patient profile. Finally, the third module defines the communication protocol for each measuring device.

**Figure 8:**
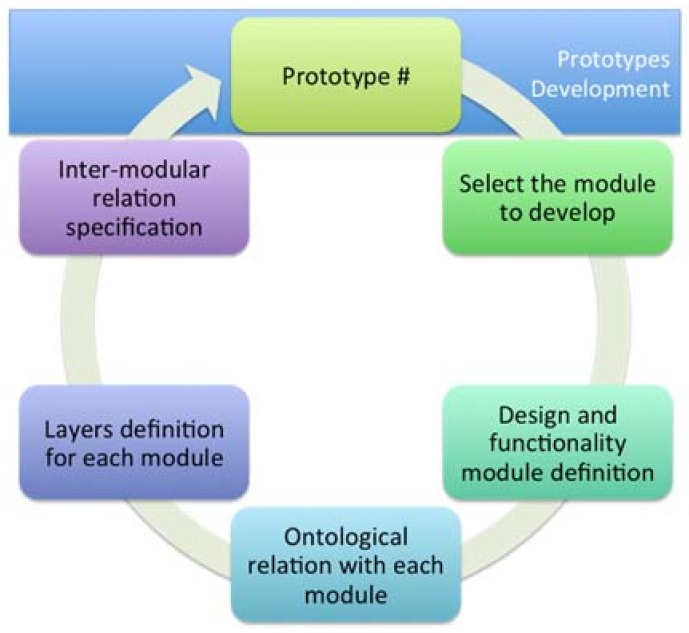
Development cycle proposed by the framework.

Furthermore, we define a development cycle that allows us to obtain different functional prototypes that define each element or module and make the final application (see Figure 8). The steps proposed by the framework for development are the following: selection of the module to be applied, definition of design patterns, definition of functional patterns, ontological relationship of each module, the layers related to their respective modules, determination of the relation between layers, integration of all elements, evaluation of the prototype, and redesign of the elements for the generation of a new prototype.

**Figure 9:**
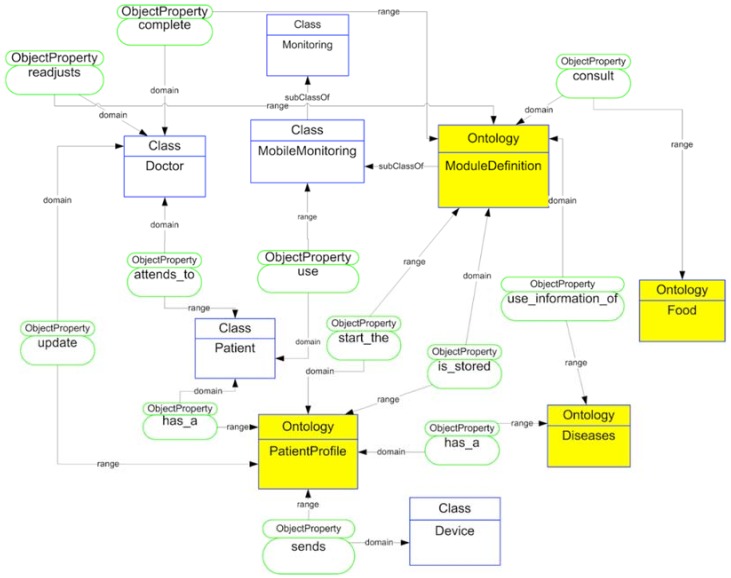
MoMOntology description.

To facilitate the development of the proposed application, we rely on an ontological model called MoMOntology (see Figure 9), presenting a classification of all possible elements to be taken into account when developing mobile solutions that can be adapted to any mobile device. To model the different services, elements and relationships that make up the framework we define each of these by means of OWL semantic web language.

We enhanced these ontologies with information about the diseases, biometrical devices, and module generation. We defined a general ontology that includes the taxonomical organization based on principal elements and the kind of data stored in augmented objects.

Depending on the information stored in the patient’s record, different modules are upgraded with new data. All activities carried out by the patient are stored on a server, making it possible for the doctor to review information from each patient.

We show the structure of the upper ontology for mobile monitoring application. The doctor and the patient are the actors who interact with the application. The patient has a profile that offers information to the application (classified under CommonProfile and IndividualProfile ontologies). This individual profile allows this module definition (MedicateTreatment, ActiveCare, and ClinicalSituation ontologies). In addition, this module definition obtains information about diseases and food ontologies. According to this classification, each of the elements is related to the initial definition of the patient profile. The definition of the modules generates the application structure for the doctor and the patient based on each of these patterns and the relationships between the modules’ definition structure.

### 4.6. Childhood Obesity Treatment

This experience allows parents to take control of the diet and physical exercise habits of their children by trying to minimize interactions with mobile devices. In this proposal we have to mention that we merged the use of NFC technology, for managing the diet information, and the smartphone accelerometer, for physical activity recognition and control [67].

**Figure 10:**
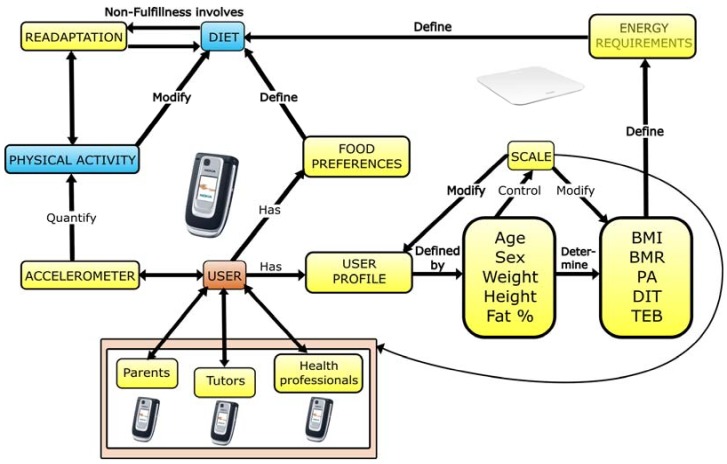
Childhood obesity treatment system scheme.

Figure 10 shows the scheme we used for the development of this proposal. The processes dealing with physical activity recognition, weight with a Wi-Fi scale, and diet are shown. Through these controls, a diet re-adaptation is possible by means of daily weight and physical exercise knowledge. In order to decide the everyday menus for children based on their energy needs, parents’ decisions are supported. Also, we kept in mind the user profile and food preferences for each child who took part in this experience.

### 4.7. Rehabilitation

The motivation of this work is that patients usually have to move to their rehabilitation center several times (even during the same week); however, sometimes factors such as lack of time and long distances affect the number of visits to their specialists. This consequently affects the quality of the rehabilitation. Moreover, some patients suffer a slight incapacitation and have to perform part of their rehabilitation at home; they may also need medical examinations to monitor their progress. The fees of well-known health care systems can be lessened by means of this kind of m-Health system.

**Figure 11:**
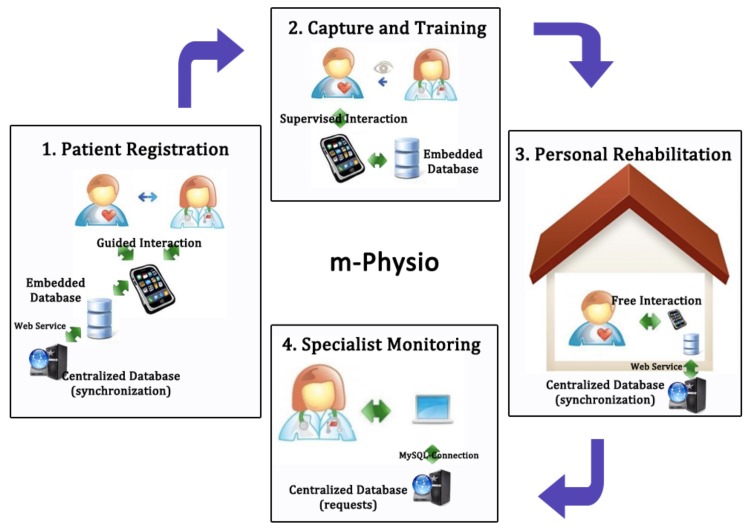
mPhysio rehabilitation process.

In Raso et al. [68] we proposed a rehabilitation system called mPhysio based on accelerometers and mobile computing. Rehabilitation specialists and their patients can use this system to improve the fulfillment of exercises and the supervision of rehabilitation tasks. We have developed this system using a mobile device and a bracelet to capture patients’ rehabilitation-relevant data. As a pre-process procedure, raw data output by mobile device accelerometer is filtered, and then the Dynamic Time Warping (DTW) algorithm is used to train and recognize movements. DTW requires simpler training and is sufficiently effective related to other pattern recognition algorithms, such as Super Vector Machine or Hidden Markov Models. Based on this recognition, patients can perform rehabilitation at home without the continuous specialist’s surveillance and can be sure of its accuracy. The mobile application captures the exercises that patients perform in their rehabilitation process and classifies them into correct, wrong (it does not match with the recognized patterns), out of time (too fast or too slow exercises), and tremor (if a tremor in the performance is detected). At any time, the specialist can consult the patient’s rehabilitation evolution by means of a web application. All of these processes are presented in Figure 11.

The proposed solution achieves the objective of ubiquitous rehabilitation performance and monitoring that enhances accuracy, is less intrusive, and reduces infrastructural needs.

### 4.8. Frailty

Frailty is a clinical syndrome related to ageing and dependence, in which detection and early diagnosis is a key indicator in improving the quality of life of elderly people [69]. However, providing a frailty assessment is currently a complicated task because of the number of factors to be taken into account for providing a comprehensive and valuable frailty diagnosis. Additionally, some factors are more important than others in frailty assessment. In this sense, functional and nutritional domains have a greater weight in the final assessment.

In this work a system was developed for supporting physicians and geriatricians in frailty detection and diagnosis, by using smartphones and accelerometer sensors in combination with a set of clinical parameters associated with frailty [70]. In addition to developing the system, this work also presents the methods and procedures needed to set up several aspects of frailty assessment in ubiquitous and healthcare environments by using a service-based approach.

Another contribution of this work is applying classification mechanisms and similarity algorithms as part of the assessment, based on studying comparisons between frail elders [71].

**Figure 12:**
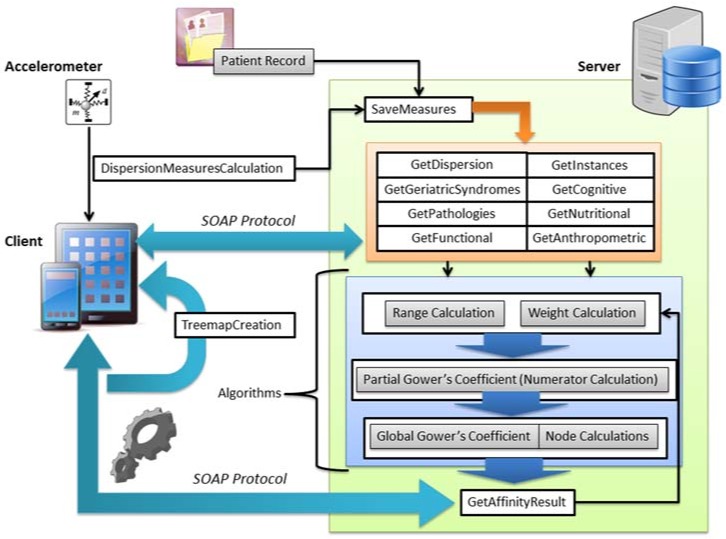
Architecture of frailty detection system.

As a part of this system, a mobile application was developed to provide the geriatrician a tool that encourages the visualization and interpretation of frailty results facilitating clinical decision-making. This mobile application provides the following functionalities: visualization of frailty variable values of patients; analysis of gait during the Tinetti test [72], modification of the weights of frailty variables to focus the analysis according to physician requirements, and visualization of frailty results. The mobile application communicates with a server, as Figure 12 shows.

Through a web service infrastructure, based on SOAP protocol [73] the mobile application gets the results associated with the web service according to the described functionalities. The algorithmic block is running in the server for obtaining the final results of the frailty of a specific patient. These results are formalized in a hierarchical tree structure and this is shown in the physician’s smartphone. The accelerometer-enabled smartphone is used to acquire accelerometer data and calculate functional variables from gait during the Tinetti test, storing these variables in the server by means of the corresponding service. The geriatrician decides the importance of each group of variables in the final frailty assessment.

This system was tested in 20 elderly patients of a nursing home by two geriatricians during a year. We concluded that this kind of system was essential for supporting the clinical decision-making of frailty because of the accuracy in the calculation of movement variables and the performance of the complete analysis, taking into account the variables from the rest of the clinical groups such as nutritional, anthropometric, cognitive, and geriatric syndromes, among others.

Traditional analysis requires more effort for frailty analysis than using this system because of the accuracy in the functional assessment (given by accelerometer sensors) and decentralization of the parameters (with traditional methods all of these are not studied together). However, it can be used as a complement of an in-depth analysis. Regarding the functional aspect, the evaluation only through Tinetti questionnaires is quite subjective; however, the use of devices increases the accuracy considerably.

### 4.9. Gait Analysis

Quantitative Gait Analysis (QGA) provides objective techniques to detect gait events, such as heel-strikes or toe-off events, which make possible to compute associated gait parameters and derived phases of the human gait cycle. Inherent gait variability in elders is characterized over time by using these gait parameters and phases, for instance, through the assessment of stride internal variability. There are currently specialized systems for QGA, which are accurate and redundant. However, these systems are expensive and they are limited to very controlled settings in clinical environments. There is a low number of gait assessments over time not reflecting the real mobility status and functional ability in subjects at their community [74] and much less being able to properly capture the inherent gait variability over time with enough sampling resolution.

**Figure 13:**
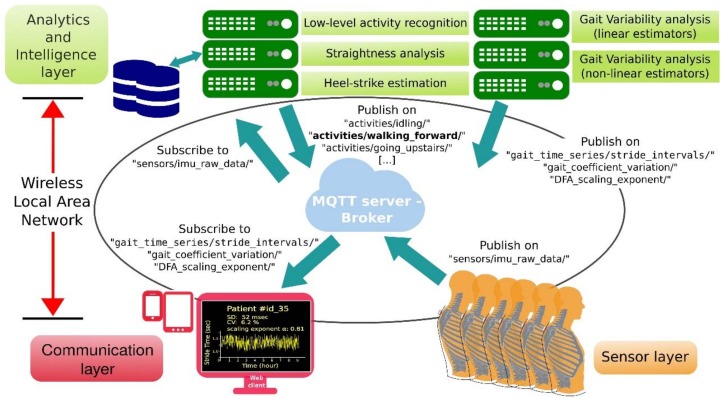
Infrastructure of gait variability monitoring system.

In this work an infrastructure to monitor the gait variability of a group of elders living in a nursing home is deployed and evaluated [75]. The infrastructure consists of a set of wearables to acquire inertial raw data from the trunk of each of the elders involved in the experiment in a non-intrusive manner. An overview of this easily deployable infrastructure created for QGA is shown in Figure 13. It is divided into three different layers: Sensor layer, Communication layer and Analytics and Intelligence layer.

**Figure 14:**
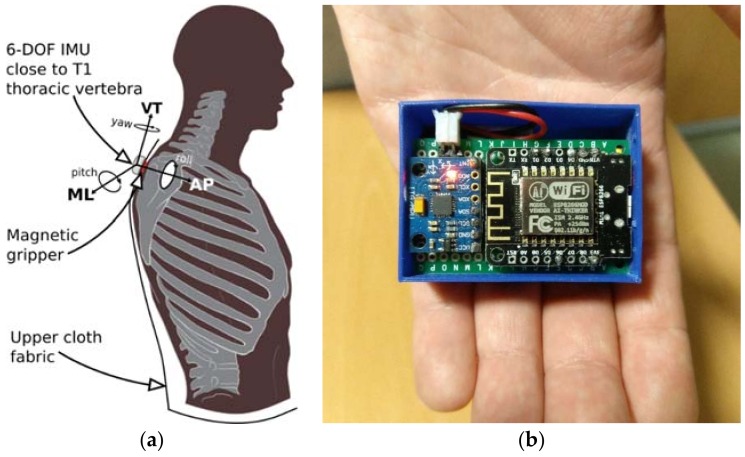
(**a**) Position of each wearable; (**b**) Hardware prototype.

The Sensor layer contains the set of wearable devices (nodes) connected to the nursing home WLAN. Each of the nodes is attached to the upper cloth of one subject, close to the T1 thoracic vertebra. A two-piece magnetic gripper is used for clamping as shown in the scheme in Figure 14a. Each wearable prototype (see Figure 14b) is equipped with a small SoC (System on Chip) which integrates a 32-bit micro-controller and an 802.11 wireless transceiver to support communication with the broker (server) connected to the same WLAN. Each wearable is also equipped with a six-Degrees-of-Freedom (6DoF) IMU. This setup allows us to acquire trunk accelerations and trunk orientations during walking for each of the elders at 50 Hz uniform sample rate and transmit them to a dedicated server (broker) using the MQTT messaging protocol.

The Analytics and Intelligence layer encloses some background services to perform offline data processing. Specifically, the low-level activity recognition service enables physical activity clustering, providing reasoning capabilities to discriminate walking forward from other low-level activities, such as running, turning, going up and down stairs or being idle. The straightness analysis and heel-strike estimation services also receive trunk accelerations and orientations. While the first considers changes in yaw rotation to segment straight gait paths within the periods of activity identified as “walking forward periods”, the second analyzes raw data gathered while walking through each of these segmented straight paths to estimate heel-strikes and toe-offs. Gait event estimation is achieved by using a modified version of the algorithm presented in [76] which helps to identify them from acceleration data through the scale-space filtering idea. Particularly, stride interval time series are derived from the heel-strike events.

Another contribution of this work is the gait variability assessment performed to 10 physically independent older adults living in the nursing home. The elders are divided into two groups of mobility subjectively scored using the Tinetti gait and balance assessment tool [67]. Five young adults also participate in the study as control group. Gait variability is automatically estimated by using two approaches: (1) a non-linear fractal analysis (detrended fluctuation analysis) which evaluates the presence of long-range correlations in the estimated stride interval time series; and (2) a statistical dispersion measure (coefficient of variation) to quantify the magnitude of the stride interval fluctuations. The impact of aging process in gait variability is studied. Stride interval variability of the elders is compared to each other and to the variability coming from the young adult group.

Despite the work does not provide conclusive results from an individual perspective, finding older adults who have less stride interval variability than younger ones. The inter-class analysis conducted shows interesting findings about the relation between the subjective characterization of gait, aging and stride interval variability estimated through the two approaches.

It should be noted that although the deployed infrastructure only has been tested under explicit gait trials by the time of this study, it is intended to be used for implicit trials and continuous gait monitoring without any required physical interaction in the residence environment. This enables long-term gait variability assessments in future steps. The communication layer illustrated in Figure 13a allows to start/stop gait trials remotely through Internet, without any physical interaction in the residence, as well as to get access (web client) to the information related with gait parameters, including gait variability.

## 5. Discussions about Lessons Learned

There are no doubts that mobile systems have transformed the way people live, work, and play. Thus, the interest of many companies in developing applications, especially for healthcare, is increasing. The mobility concept allows patients to access medical information when and wherever they want. For these reasons, it is necessary to lead the healthcare change for reducing costs, proving outcomes, and increasing access [77]. Also, it is important to mention that we are in an early stage of m-Health. Regarding the European Union e-Health Task Force Report, “users need not only understand the possibilities of e-Health tools, but they have control over how they interact with them” [78].

Regarding m-Health experiences detailed in this work, Table 3 shows a summary of relevant features considered by all the systems presented.

The proposed approaches from the MAmI Research Lab mainly focus in terms of improving healthcare tasks by minimize interaction with devices and taking advantages of mobile phone features, in conjunction with other sensing capabilities that can be provided by wearable devices or body-worn sensors.

Looking closely to the Table 3, aspects such as physical exercise or vital signs controls, are generally contemplated by several of the developed m-Health applications. As we have mentioned previously, the user interaction is another important aspect in those systems in which feedback to the user is mandatory, however we do not want to distract the user’s attention and still get continuous monitoring.

Other aspects, such as remote health care and tele-monitoring are recurrent in the systems presented. However, there are missing concepts, like connections with similar solutions or sharing users’ experiences. Thus, it is important to look for guidelines in order to achieve m-Health applications focusing on all mentioned aspects. In this way we can develop widely used m-Health solutions for covering all concerns.

**Figure 15:**
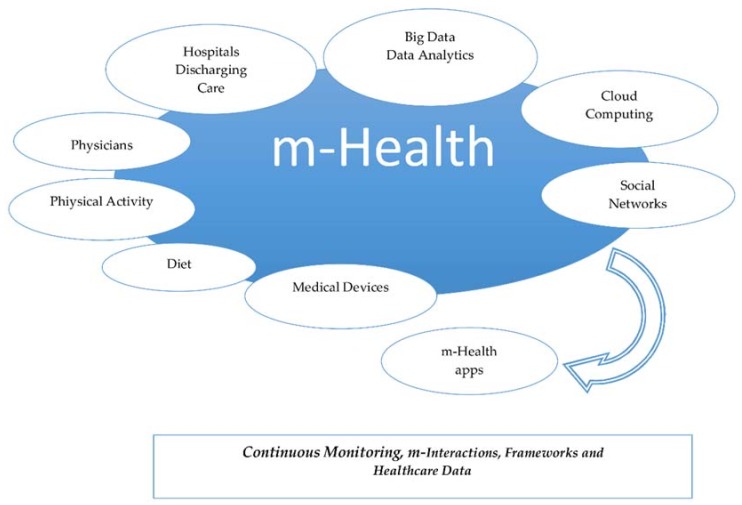
m-Health Transformation.

We propose an approach to these guidelines in the following graph (see Figure 15). The first argument is the behavioral change of moving health care out of hospitals. Thus, a discharge of caring services results in better attention because of the reduction of crowded hospitals; health care would be provided everywhere. Thus, new opportunities for physicians related to monitoring and remote caring of patients are appearing.

There are many m-Health applications available that facilitate patient access to medical information. However, most people prefer direct communication with physicians [79]. Despite this, a large percentage are interested in m-Health applications. Thus, it is very important to improve these applications in order to get the most users’ attention and change their health view. It is also important to involve others user roles, such as hospital staff members and, especially, physicians. Actually, there is a lack of communication because these apps are developed only for end users. The participation of all members of the health community should be desirable, perhaps thought the integration of gamification elements to promote engagement and empowerment to users.

Furthermore, the trend in framework development, in order to generalize the use and the own development of the m-Health applications, has to be considered. For example, in terms of monitoring, providers of vital signs devices have to open communication protocols to the development commmunity. In this manner, frameworks and applications can receive data from each device and operate according to diseases and patient profile. For example, in Diabetes disease monitoring, big companies like Dexcom and projects such as Nightscout have begun to provide mechanisms to achieve this.

On the other hand, the need for easier interaction must be considered. We do not have to distract the attention of the user by inserting data into the apps. The tendency should be to develop applications that run in the system background by promoting simple and direct awareness and avoiding time-consuming or extended user interaction. In this case, we can talk about “disappearing interaction.” For example, the inertial measurement unit, embedded into smartphones or wearable’s to monitor the time spent walking per day, makes it possible for the application to run during the performance of physical activities. Thus, users can take advantage of using these features to change their wellbeing and health habits.

Another important aspect to consider is diet. We need to look for easy new interactions trying to control the food intake, calories consumption, carbohydrates, fats, etc. In this manner, get healthy lifestyle habits through a balanced diet will be possible.

Among current trends, Cloud computing enables the storage of the health care services in a secure location that can be accessed anywhere. It provides platforms for data synchronization collaboration and participation in heath information ecosystems and advanced capabilities. Networking between hospitals and physicians is also possible.

Data Analytics is another issue that has to be addressed highlighting the importance of Big Data (Big Data Analytics). We need to realize that prevention is better than cure. In this sense, hospitals and other Health institutions, should have the responsibility for uploading patient records to the Cloud in a secure and privacy way to facilitate the performance of data analysis and getting patterns from the users while they are involved into their daily activities (physical, mental, food, sleep, etc.). In this sense, Big Data Analytics can provide useful information in order to find patterns that will be the base for a diagnosis or discover a tendency to suffer from a disease.

All of the above mentioned is in consonance with the increasing relevance of Big Data in Healthcare to predict epidemics and diseases, achieving a better quality of life and avoiding deaths. Thus, because of the population is increasing and living longer it is necessary to change the way of thinking in Healthcare Data analysis for decision making. It is obvious that physicians must rely on large amount of data offered by Big Data systems as an essential complement of their own experience and professional opinion. However, new policies in the treatment of medical record privacy and security have to be addressed. Similarly, health authorities should be aware to share their health records in the Cloud. At this way, Cloud and Fog Computing joining with Big Data Analytics could become a reality.

Social networking allows to compare costs and physician practices, as well as obtaining advice about treatments and support of living with chronic conditions. Sharing information with peer groups also promotes the adoption of m-Health solutions by means of a more effective communication.

Continuous monitoring is another important aspect of m-applications that is growing in scale by managing chronic diseases. Individualized data analytics can be obtained to ensure wellness and dealing with prevention aspects. Patients can compare costs of different providers, diagnosis support, and personalized medicine. In this sense, it is important to mention that continuous monitoring is an advantage of the m-Health proposals. Patients and physicians can collect continuous health data to promote monitoring and prevention.

**Figure 16:**
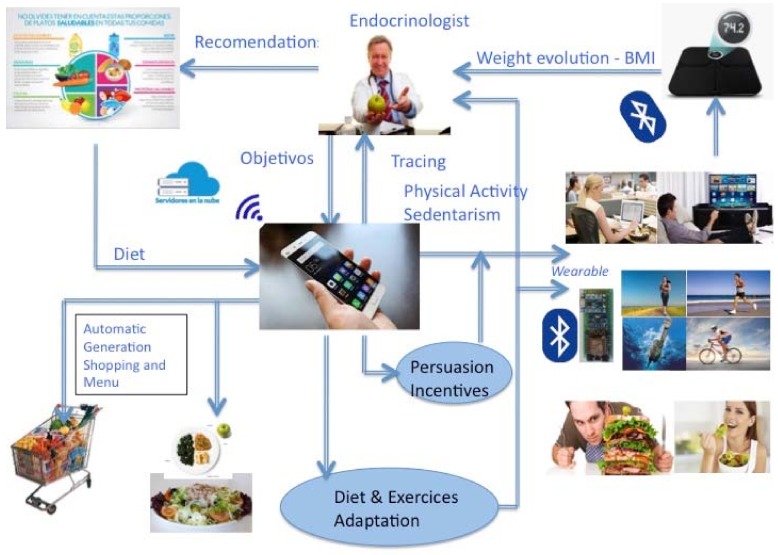
Obesity Prevention through Wearables.

At this point, a new insight of continuous health monitoring for prevention can be addressed. That is possible to the sensor growing embedded into daily objects, that is, wearable’s for health. Figure 16 shows a new proposal for obesity prevention that considers all the above-mentioned aspects. Thus, a recommendation for all the wearable manufacturers has to be considered. That is, they have to assume policies to promote the continuous data uploading to the Cloud. The increase of healthcare data can be considerable if the consumers allow uploading their continuous monitoring data, in an easy and transparent manner, in their own benefit.

Currently, this vision needs more time to become real. Although there are many m-Health applications, users doubt about the efficiency of this kind of applications. In fact, it is not difficult to evaluate how many times an application has been run. Perhaps, as we mentioned before, it is necessary to involve physicians when patients are running this kind of applications, even encouraging its use through gamification techniques. Also, communication between patients and physicians is very important and necessary, and this communication should be transparent for the patients. Physicians would receive monitoring data in an adequate format, in order to make the corresponding decisions for each case.

Regarding our area of expertise, and considering the literature in m-health and the MAmI Lab m-experiences during the last ten years, we have learned the following aspects. First, we have to create the right conditions so the hospital staff does not worry about the use of mobile devices. Today, this use is undervalued in hospital environments. For this reason, we think a meaningful way to overcome this obstacle is by embedding sensors into mobile devices. From the MAmI Lab, we adapted NFC technology to allow physicians and nurses to manage information by only touching NFC tags with the smartphone. For that, it is necessary to tag the context information of the hospital adequately. Another concern involves the use of frameworks. We have developed the project called MoMo, for getting information from medical devices and adapting this information to each disease and patient profile. Finally, and thanks to the inertial measurement unit embedded into the mobile devices, we can monitor physical activity. Furthermore, and due to gait analysis recognition, elderly patients can be monitored in terms of frailty or dementia. Once again, the interaction “disappears” because the users only have to wear their smartphones on their waists. However, our m-Health prototypes could be improved, taking into account all the above-mentioned aspects.

## 6. Conclusions

In this work, we have addressed several perspectives on m-Health research. These perspectives include the studied literature regarding m-health and the acquired experience developing projects for m-health during the last ten years. From our point of view, it is necessary to have a new way of conceiving this sight in order to complement the traditional healthcare services at hospitals, home, work, etc. Thus, it is important to take into account relevant aspects in terms of m-Health. The following aspects are the main challenges to face in m-health, obtained from our m-experiences. These challenges can be summarized as follows: continuous monitoring, full involvement, interoperability and disappearing interaction. Firstly, we consider the idea of continuous monitoring that will provide physicians with a new dimension of patient data. Thus, it will be possible to study the evolution of particular disease in each patient. Secondly, it is mandatory to transform m-Health applications as a complement to hospital healthcare services. Thus, the application data has to be managed by patients and physicians. Third, the integration of Cloud Computing and social networks strives against patient isolation. Finally, the interaction must be closer to patients and physicians.

Moreover, from the development of the detailed m-Health systems, we have studied some of the previously mentioned assumptions. First, the idea of involving physicians and patients in our multi-monitoring approach has been presented. In addition, an easier interaction proposed achieves adequate levels of success in this kind of applications. Following all of the above, the proposals of gait analysis and activity recognition by means of accelerometer-enabled smartphones are crucial in our last approaches.

Finally, a new insight for m-Health arises since Cloud and Fog Computing joining with Big Data Analytics have appeared into the healthcare landscape. On this basis, a new conception of continuous monitoring and real-time assessment of healthcare emerges. This model uploads health data to the Cloud for further processing in a transparent manner or, in other cases, it decides in a dynamic manner whether process selected data locally in the mobile phone (Fog computing). After that, we can all benefit from Big Data Analytics for achieving a preventive medicine instead of curative one.

## Figures and Tables

**Table 1:** Summary of each system and domain.

System Domain	Description	Pros	Cons
Nursing Care	NFC system for nursing care at hospitals	Reduce burden of nurses in managing patient records	Nurses require a previous knowledge of the system
Nursing Training	NFC system to improve training of nurses	Help nursing students to simulate clinical tasks using new technologies	Students require a previous knowledge of the system and clinical procedures
Alzheimer caregivers’ support	NFC system to help caregivers at Alzheimer’s day center	Help caregivers to manage tasks, incidences and reminders at a day center	Incidences should be manually registered by caregivers according a limited list.
Mobile prescriptions	System based on NFC and desktop applications to manage medications and prescriptions	Automate the process of requesting medicines from home, in elderly people.	Current social services do not offer mechanisms to take home medicines (at least in Spain)
Vital signs multi-monitoring	Framework to help in developing of mobile apps focused on monitoring of vital signs	Facilitate the generation of mobile apps for healthcare following several processes	Framework should be known in detail to develop useful apps
Childhood Obesity Treatment	NFC system to monitor diet and physical exercise by parents of children with overweight problems	Acquire information from different domains to analyze issues based on personalized profiles	Food and meals should be inserted manually by parents to monitor this aspect
Rehabilitation	Mobile system to enhance the monitoring of rehabilitation tasks at home	Provide a mobile tool to continue with rehabilitation tasks at home based on remote clinical support	This tool depends on internet connection for a real-time monitoring and iPhone. It is only useful as a clinical complement
Frailty	Mobile system to support clinicians to make a diagnosis based on frailty index (in seniors)	Provide a relative frailty index to facilitate diagnostics considering all clinical domains	Functional domain is not analyzed exhaustively (same importance than the rest of domains)
Gait Analysis	System based on wearable sensors to analyze gait of elderly people in a quantitative way, in a nursing home	Accuracy of the system and ease of communication with other devices/apps	Wearable sensor should be placed in the correct position to avoid data variability

**Table 2:** NFC tags content.

**(a) Tasks**			
**Task**	**Place**	**Tag**	**Process (awareness)**
Patient monitoring	Room	Patient	Blood pressure/Pulse
Drugs control	Room	Patient	Drug checks
Diet	Room/Nurse desk	Patient/Bed/Interactive display	Select and modify
Care	Room	Patient	Care protocol
Analyses control	Room/Nurse desk	Patient/Bed/Interactive display	Select
Managing information server	Nurse desk	Interactive display	Storing information
Patient record for nursing	Nurse desk	Interactive display	Getting information
Preparing drugs & doses	Drugs cabinet	Meds	Doses
**(b) Collaboration**			
**Users**	**Place**	**Tag**	**Process (awareness)**
Nurse—Physician	Room/Nurse desk	Patient/Bed/Interactive display	Control of blood, urine tests, drug, diet/Monitoring
Nurse—Nurse	Nurse desk	Interactive display	Shift change
**(c) Tag Content**			
**Tag**	**Content**		**Awareness**
Patient	Drugs & doses (last time)/Blood & urine tests (time, results)/Diets/dressing/Monitoring/Contraindications of medication		Yes
Bed	Patient Id./Pending blood & urine tests/Diet		Yes
Room	Patient Id.		Yes
Meds	Active principle/Contraindications/Doses		No
Interaction	(commands) Control/Select/Zoom/Back/…		Yes

**Table 3:** Summary of features from developed m-Health systems.

MAmI m-Health Features	Care Complement	Physical Exercise	User Experience	Similar Solutions	Vital Signs Control	Remote Health Care	User Interaction
Nursing Care & Nursing Training	√	X	X	X	√	X	X
Alzheimer Caregiver’s Support	√	√	X	X	X	X	X
Mobile Prescriptions	√	X	X	X	X	X	X
Vital Signs Multi-Monitoring	√	X	X	X	√	√	√
Childhood Obesity Treatment	√	√	X	X	√	X	√
Rehabilitation	√	√	X	X	X	X	√
Frailty	√	√	X	X	√	√	√
Gait analysis	√	√	X	X	X	√	√
